# Phytate‐iron molar ratio and bioavailability of iron in Bangladesh

**DOI:** 10.1111/tmi.13750

**Published:** 2022-04-19

**Authors:** Sabuktagin Rahman, Nazma Shaheen

**Affiliations:** ^1^ Public Health, School of Medicine Griffith University, Gold Coast Campus Southport Queensland Australia; ^2^ Institute of Nutrition and Food Science University of Dhaka Dhaka Bangladesh

**Keywords:** Bangladesh, bioavailability, iron, molar ratio, Phytate

## Abstract

**OBJECTIVE:**

Phytate, an important component of plant origin foods, works as a chelator for mineral nutrients such as iron. Estimating the phytate‐iron molar ratio is a traditional method to assess the bioavailability of dietary iron, and a ratio >1 is suggestive of poor absorption of iron through the intestinal mucosa. In Bangladesh, the ratio is considerably higher; nonetheless, the haemoglobin and ferritin status are satisfactory. Hence, we appraised phytate‐iron molar ratios and concomitant haemoglobin and ferritin status.

**METHODS:**

Dietary intake of iron and phytate was estimated in non‐pregnant non‐lactating women and school‐age children from a nationally representative survey. The phytate‐iron molar ratios were estimated. Linear regressions on haemoglobin for the phytate‐iron molar ratios and on molar ratios predicting inflammation‐adjusted ferritin were performed.

**RESULTS:**

The median ratios were 6.12 in women and 5.47 in children, with corresponding haemoglobin concentrations of 12.6 and 12.5 g/dl. Hypothetical lowering of the ratios by ~50% revealed a nominal increment of haemoglobin and ferritin.

**CONCLUSION:**

The standard cut‐off phytate‐iron molar ratio of >1 is inconsistent with the iron and haemoglobin status of the Bangladeshi population. One plausible explanation for the inconsistency is a non‐dietary environmental factor—groundwater iron. Isotope studies incorporating the iron from dietary and the drinking groundwater sources are needed to establish a ratio which might better explain iron bioavailability.

## INTRODUCTION

Anaemia, defined as a haemoglobin level below a specified cut‐off, is a major public health problem in low‐ and middle‐income countries [1]. Iron deficiency (ID) is considered the most common cause of anaemia [2]. Hence, dietary iron and its bioavailability is an important aspect in the genesis of anaemia. Dietary iron is sourced from animal and plant foods. Amongst plant foods, phytate, an inositol hexaphosphate, is the primary storage form of both phosphate and inositol [3]. It is endowed with beneficial roles as an antioxidant and anticarcinogen [4]. Owing to its ability to bind and chelate minerals, phytate can decrease the bioavailability of critical nutrients such as zinc, iron, calcium [5] and magnesium [6]. The traditional diet in Bangladesh is cereal based; nearly 70% of calories come from the consumption of rice [7]. A nationally representative micronutrient status survey revealed that >90% of the total intake of phytate come from phytate‐rich cereals (National Nutrition Service, Bangladesh Personal Communication). Phytate inhibits the absorption of key nutrient minerals such as iron, thus hindering their bioavailability. A commonly used method to assess the bioavailability of iron is by measurement of the phytate‐iron molar ratio, calculated as the ratio of intake of phytate (mg/day) relative to its molecular weight to the intake of iron (mg/day) relative to its molar weight [8, 9]. A phytate‐iron molar ratio >1 implies inhibited bioavailability of iron [10]. In Bangladesh, the ratio is higher than the cut‐off; it is 12.8 in pregnant women [9] and 4.12 in female residential university students [11]. However, against this backdrop of low bioavailability of dietary iron, the national estimated values of haemoglobin and inflammation‐adjusted ferritin are adequate [12]. As per the national micronutrient status survey 2011–2012, the prevalence of anaemia in school‐age children (6–14 years) was 17.1%–19.1% (according to the age subgroups) and 26% in non‐pregnant women (15–49 years). The prevalence of ID in these populations was 3.9%–9.5% (according to the age subgroups) and 7.1%, respectively [12]. In a trial of antenatal supplementation of multiple micronutrient supplements versus iron‐folic acid supplementation in a northwestern Bangladeshi district, the baseline prevalence of ID in pregnant women was 4% [13]. In adolescent girls (9–13 years) in a northwestern district, the prevalence of ID was 0.5% [14]. The low prevalence of ID in Bangladeshi populations has been linked to the ingestion of iron from groundwater as a source of drinking water, which contains variable, often large amounts of bioavailable iron [12, 13, 15, 16, 17]. Therefore, to advance the understanding of iron bioavailability, the present analysis examined phytate‐iron molar ratios and the concomitant haemoglobin and ferritin status in the Bangladeshi population.

## METHODS

The data set of the national micronutrient status survey 2011–2012 was used, which reported the biochemical status of key micronutrients in the Bangladeshi population and measured the quantitative intake of dietary nutrients. The study populations were non‐pregnant non‐lactating women of reproductive age (NPNLW, 15–49 years) and school‐age children (SAC) aged 6–14 years.

### Dietary intake estimation

Dietary intake was assessed by a validated 7‐day semi‐quantitative food frequency questionnaire comprising 53 commonly consumed Bangladeshi foods [18]: various cereals, legumes, leafy vegetables, non‐leafy vegetables, small fish, large fish, chicken, beef, mutton, eggs, milk, etc. For each item, the amount consumed over the preceding 7 days was recorded. An updated FCT on Bangladeshi foods was used to calculate the nutrient intakes [19]. For a few nutrients that were missing in the FCT, the USDA database on the nutrient values was used [20]. Edible portion coefficients for Bangladeshi foods were used to derive the edible amount [19]. Cooked food amounts were converted into the raw food weight by dividing by the appropriate yield factors [19]. Nutrient values were calculated per 100 g of the raw weight of consumption as per the indication in the FCTs. Iron and phytate consumption was estimated per day per 100 g of raw food according to the food composition tables [19, 20, 21]. The details of the methods are provided in Rahman et al. [18]

### Phytate‐iron molar ratio

Using the quantitative intake data of the study populations, the molar ratio of phytate to iron was calculated [8, 9, 11] as: $\left[\text{Intake of phytate}\;\left(\mathrm{mg}/\mathrm{day}\right)/660\right]/\left[\text{Intake of iron}\left(\mathrm{mg}/\mathrm{day}\right)/56\right].$

### Measurement of Hb and ferritin

Hb was assessed by using a HemoCue®Hb 301 system on venous blood (HemoCue AB, Angelholm, Sweden). Serum ferritin and inflammation adjustment biomarkers such as C‐reactive protein (CRP) and 1‐α‐acid glycoprotein (AGP) were measured by sandwich ELISA.

### Statistical analysis

Linear regression was performed with the phytate‐iron molar ratio (x‐axis) predicting the haemoglobin concentration (y‐axis). Slopes were appraised against the specified percentiles of the phytate‐iron molar ratio and haemoglobin concentrations to assess the consistency of the ratio and iron bioavailability. Serum ferritin was adjusted for inflammation by the correction factor approach as described by Thurnham et al. [22] Linear regression was performed to assess the association of phytate‐iron molar ratio and inflammation‐adjusted ferritin. Data were analysed using STATA 13 statistical software (STATA Inc., College Station, Texas, USA).

### Data acquisition and the ethical approval

Permission to use the data was obtained from the National Nutrition Services (NNS), Ministry of Health, Government of Bangladesh. Ethics approval was obtained at the Institutional Review Board, icddr,b, Bangladesh. Primary data were collected after obtaining written informed consent from the participants.

## RESULTS

The estimated phytate‐iron molar ratio was 6.12 in NPNLW and 5.47 in school‐age children. Table 1 shows a linear association of phytate‐iron molar ratio, and haemoglobin concentration was observed in NPNLW (coefficient − 0.04, *p* = 0.05) and similarly in SAC (coefficient − 0.018, *p* = 0.009).

**TABLE 1:** Linear regression shows an association of phytate‐iron molar ratio and haemoglobin concentration in non‐pregnant non‐lactating women (NPNLW) and school‐age children (SAC)

	Constant	Coefficient	Standard error	*P* value	95% CI
Non‐pregnant women	12.82	−0.04	0.02	.05	−0.08, 0.001
School‐age children	12.59	−0.018	0.05	.009	−0.03, −0.004

**FIGURE 1:**
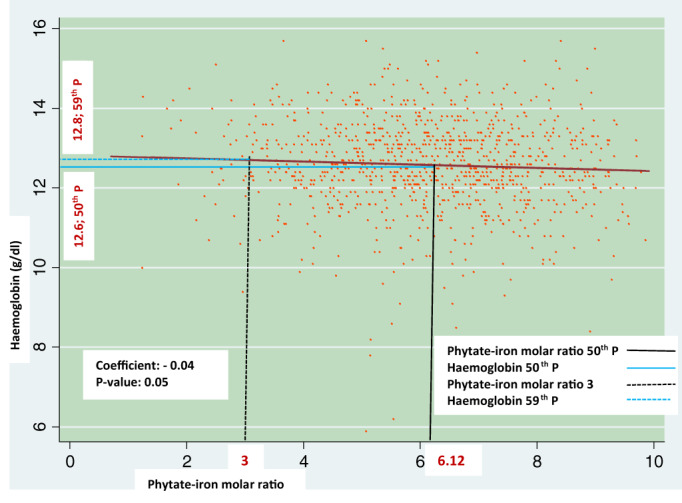
Graphical depiction of the association of phytate‐iron molar ratio and haemoglobin concentration in NPNLW

Figure 1 graphically depicts the linear association of phytate‐iron molar ratio and haemoglobin concentration in non‐pregnant non‐lactating women. The phytate‐iron molar ratio at the 50th percentile was 6.12; the corresponding haemoglobin value (at 50th percentile) was 12.6 g/dl. Figure 1 further illustrates that the haemoglobin concentration was 12.8 g/dl and corresponded to a hypothetical phytate‐iron molar ratio of 3.

**FIGURE 2:**
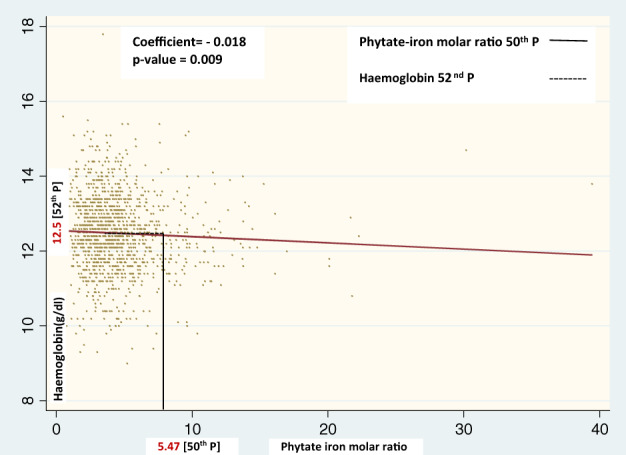
Graphical depiction of the association of the phytate‐iron molar ratio and haemoglobin concentration in school‐age children (SAC)

Figure 2 graphically depicts the linear association of phytate‐iron molar ratio and haemoglobin in children aged 6–14 years. The phytate‐iron molar ratio at the 50th percentile was 5.47, which corresponded to a haemoglobin concentration of 12.5 g/dl at the 52nd percentile.

The phytate‐iron molar ratio and inflammation‐adjusted ferritin showed no statistically significant association in SAC (coefficient: −0.28, *p* = 0.77) and NPNLW (coefficient: −0.26, *p* = 0.80) (Table 2).

**TABLE 2:** Linear regression shows the association of the phytate‐iron molar ratio and the inflammation adjusted ferritin* in children (SAC) and women (NPNLW)

	Constant	Coefficient	Standard Error	*P* value	95% CI
SAC	65.55	−0.28	0.99	.77	−2.23, 1.66
NPNLW	65.32	−0.26	0.99	.80	−2.2, 1.69

* Correction factor approach [22].

## DISCUSSION

Using nationally representative data, we attempted an appraisal of the estimate of phytate‐iron molar ratios and the corresponding haemoglobin and ferritin concentrations in Bangladeshi women and school‐age children against the standard cut‐off value of the phytate‐iron molar ratio that quantifies the bioavailability of iron.

In NPNLW (Figure 1), the median phytate‐iron molar ratio of 6.12 was considerably above the standard cut‐off value of 1, indicating the inhibition of the absorption potential of iron. The corresponding concentration (50th percentile) of haemoglobin (12.6 g/dl) was higher than the cut‐off defining anaemia in NPNL women (12 g/dl) [23]. The estimate of haemoglobin is complemented by a satisfactory level of inflammation‐adjusted ferritin (63.7 ng/mL; results not shown) which is several fold above the cut‐off defining ID (15 ng/mL) in women [24]. Figure 1 further shows that if the phytate‐iron molar ratio is hypothetically reduced to 3, it incurs only a small increase in haemoglobin concentration (12.8 g/dl). Similarly, Galetti et al. [25] found a nominal haemoglobin increment in women from a pooled analysis of multiple data sources [25], and that the fractional absorption of iron was responsive to the ferritin status up to a ferritin level of 51 ng/mL. This implies that, as the ferritin value increases (i.e., upto 51 ng/mL), the fractional absorption of iron decreases steadily. At a ferritin level >51 ng/mL, the fractional absorption of iron plateaus [25]. Since the inflammation‐adjusted ferritin in women in the present study was 63.7 ng/mL, we assume that the absorption of iron was stable; that is, inhibition of absorption of iron had probably set in. This possibly explains the small increment of haemoglobin if the phytate‐iron molar ratio of 6.12 is hypothetically lowered to 3. According to the standard cut‐off value for the phytate‐iron molar ratio, the estimate of 6.12 or even 3 is not consistent with the efficient absorption of iron. Interestingly, haemoglobin and ferritin levels in the subjects were satisfactory.

In SAC, similar to the observation in women, the median (i.e., 50th percentile) phytate‐iron molar ratio of 5.47 was higher than the cut‐off of 1. At this percentile of the ratio, the corresponding haemoglobin was 12.5 g/dl (52nd percentile) and thus considerably above the cut‐off defining anaemia in this age group (12 g/dl) [23]. The regression slope clearly demonstrates that hypothetical lowering of the phytate‐iron molar ratio to 1 or less would increase the haemoglobin estimate only marginally (Figure 2), further indicating low‐grade absorption of iron.

In SAC, the inflammation‐adjusted ferritin was 63.8 ng/mL (results not shown) which was consistent with sufficient iron status. Linear regression (Table 2) of the phytate‐iron molar ratio and inflammation‐adjusted ferritin showed no association (*p* = 0.77). The intercept (65.55) and the slope (−0.28) imply that if the phytate‐iron molar ratio was hypothetically decreased to 3 (i.e., ~50% reduction), inflammation‐adjusted ferritin would be ~64.5 ng/mL. The level of ferritin at the molar ratio 3 relative to the intercept value of 65.5 ng/mL ferritin signifies a negligible change(~1 ng/mL) and a tiny effect of dietary iron on serum ferritin status in a state of considerable hypothetical dietary improvement.

In NPNLW, inflammation‐adjusted ferritin was 63.7 ng/mL (results not shown). There was no linear association of the ratio and inflammation‐adjusted ferritin (*p* = 0.8). The intercept (65.32) and the slope (−0.26) imply that if the phytate‐iron molar ratio was hypothetically decreased to 3 (i.e., >50% reduction), the inflammation‐adjusted ferritin would be ~64.51 ng/mL. This is a tiny increment and further confirms the poor potential of dietary iron to ferritin status.

At the population level, the weak influence of dietary iron even after a hypothetically improved diet to affect serum iron status is crucial policy information. It underscores the dominant, intractable role of groundwater iron in maintaining the population iron status in Bangladesh. This merits a cautionary approach to wide‐scale blanket iron supplementation for the targeted population groups with standard iron doses, since in an iron‐replete state, additional supplemental iron might be counterproductive and may result in side effects [26, 27]. However, in some parts of the country where the concentration of water iron is particularly low, maintenance of the supplemental iron programmes for pregnant women and young children remains necessary, preferably in low doses.

Absorption of iron (i.e. inflammation‐adjusted ferritin) from combined groundwater and dietary iron sources was low and nearly identical in children and women. The possible reason for low iron absorption in the presence of high iron status is difficult to ascertain. But, we assume that hepcidin (not measured in the study), which downregulates the absorption of iron at the basolateral intestinal mucosa in the presence of high body iron status, might be implicated in the inhibition of iron absorption [28]. The other possible reason for hepcidin‐induced inhibition of iron absorption is the presence of inflammation and infection. However, the setting is non‐endemic for malaria, and the presence of common infection and/or inflammation as measured by the CRP > 5 mg/L was 4.9% (school children) and 9.5% in non‐pregnant non‐lactating women [29] which is an acceptable level for a developing country. Moreover, the potential effect of inflammation was adjusted to report the inflammation‐adjusted ferritin status in the present study. Therefore, the following steps plausibly occur: First, ingestion of iron through groundwater which has an average absorption of 23% on an empty stomach [30]. Second, this might lead to a high body reserve of iron. Third, this is likely to be responsible for the decrease in the efficiency of absorption of iron (plausibly hepcidin induced) at the estimated phytate‐iron molar ratios. This highly absorbable iron may have accumulated as a sufficient reserve in the body through drinking groundwater since the early months of life, and the efficiency of absorption declines with age.

The low potential of iron bioavailability mimics the Indian setting. Indian studies observed that in non‐anaemic women whose staple diet was phytate‐rich rice, the absorption of iron is as low as 2.7 ± 1.7% [31] and 7.3 ± 5.9% [32]. The Indian studies did not report the iron from drinking water, and the low absorption might be diet induced (i.e., phytate). In contrast to India, in Bangladesh, much of the fair status of iron in the population is explained by drinking iron‐rich groundwater [16, 26], and this might implicate the low bioavailability of iron.

The intake of highly bioavailable water iron [30] also explains the discrepancy between the estimated dietary phytate‐iron molar ratio and the iron/haemoglobin status in the population. The inhibitory factors of phytate are removed by washing with water, that is, when groundwater is mixed with food. Phosphates are formed from phytates during enzymatic dephytinisation leading to a phytate fraction with a small residual inhibitory effect on water iron absorption [33]. Nonetheless, most of the water is drunk on a relatively empty stomach, leaving groundwater iron absorption unhindered.

A hypothetical change of the phytate‐iron molar ratio from 6.12 to 3 (Figure 1) translates into a massive change in the dietary pattern. To enable this, consumption of rice, responsible for 90% of the phytate in the diet (personal communication, National Nutrition Services, Bangladesh), would have to be reduced by half and replaced by food free of phytate, that is, a ~25% increment of intake of food from animal sources (ASF) (calculations not shown). Despite this profound hypothetical modification of the diet to bring the phytate‐iron molar ratio closer to the 1 point, which corresponds to the state of more efficient absorption of iron, the increment in haemoglobin would be modest in the setting. This suggests that against a background of a high body iron level, inhibition of iron absorption possibly sets in at a higher ratio than the traditional cut‐off. This challenges the validity of the standard cut‐off of the ratio (i.e., 1) in Bangladesh. Besides, such a massive modification of the diet is unlikely because of the proportion of rice in calories consumed [34] and the concomitant dietary phytates in the Bangladeshi diet.

It is difficult to infer whether the inhibition of the absorption of iron sets in at the ratio of 6.12 (50th percentile in NPNLW) or at 5.47 (50th percentile in SAC). The estimate of the ratio could be within the range of 3 (i.e., when intake of phytate is lowered by~50%) and 6.12 or around this margin. But, it is unlikely to be <1. A well‐designed isotope study is needed in the setting to precisely estimate the phytate‐iron molar ratio which could better explain the iron bioavailability.

One limitation of our study is that the SQFFQ tool we used did not validate the dietary phytate. However, it measured and validated the dietary fibre, and phytate is a principal component of dietary fibre [35]. Both dietary fibres and phytates are associated with significant chelating properties of mineral absorption. Our SQFFQ has shown that the tool was adequately valid to measure dietary fibre (only 4.7% of observations falling into the extreme opposite quintile; a moderate kappa agreement statistic of 0.42 and Lin's concordance of absolute agreement 0.62) [18]. Despite the fair degree of validity with the measurement of dietary fibre, not measuring phytate and confirming the validity only in children constituted a weakness.

## CONCLUSION

The standard cut‐off value of >1 for the phytate‐iron molar ratio that marks inefficient absorption of dietary iron does not conform with the population‐level iron and haemoglobin in Bangladesh, which exhibit good status. In Bangladesh, iron status is predominantly influenced by groundwater iron with a minimal role of dietary iron. The dynamics pertaining to iron intake sources in Bangladesh and iron absorption characteristics reveal that the bioavailability of iron remained functional at a higher than standard phytate‐iron molar ratio, but not far off reaching the inhibition point. A well‐designed isotope study incorporating dietary and drinking water iron is required to establish a new ratio of phytate to iron in this and similar settings, which might better explain iron bioavailability.
